# Lean mass and lower limb muscle function in relation to hip strength, geometry and fracture risk indices in community-dwelling older women

**DOI:** 10.1007/s00198-018-4795-z

**Published:** 2018-12-14

**Authors:** A. Elhakeem, A. Hartley, Y. Luo, A. L. Goertzen, K. Hannam, E. M. Clark, W. D. Leslie, J. H. Tobias

**Affiliations:** 1 Musculoskeletal Research Unit, Translational Health Sciences, Bristol Medical School, University of Bristol, Bristol, UK; 2 MRC Integrative Epidemiology Unit, Population Health Sciences, Bristol Medical School, University of Bristol, Bristol, UK; 3 Department of Mechanical Engineering, University of Manitoba, Winnipeg, Canada; 4 Department of Radiology, University of Manitoba, Winnipeg, Canada; 5 Department of Internal Medicine, University of Manitoba, Winnipeg, Canada

**Keywords:** DXA, Fracture risk index, Jumping mechanography, Physical performance, Sarcopenia

## Abstract

**Summary:**

In a population-based sample of British women aged over 70 years old, lean mass and peak lower limb muscle force were both independently associated with hip strength and fracture risk indices, thereby suggesting a potential benefit of promoting leg muscle strengthening exercise for the prevention of hip fractures in postmenopausal women.

**Introduction:**

To investigate cross-sectional associations of lean mass and physical performance, including lower limb muscle function, with hip strength, geometry and fracture risk indices (FRIs) in postmenopausal women.

**Methods:**

Data were from the Cohort of Skeletal Health in Bristol and Avon. Total hip (TH) and femoral neck (FN) bone mineral density (BMD), hip geometry and total body lean mass (TBLM) were assessed by dual x-ray absorptiometry (DXA). Finite element analysis of hip DXA was used to derive FN, intertrochanteric and subtrochanteric FRIs. Grip strength, gait speed and chair rise time were measured objectively. Lower limb peak muscle force and muscle power were assessed by jumping mechanography.

**Results:**

In total, 241 women were included (age = 76.4; SD = 2.6 years). After adjustment for age, height, weight/fat mass and comorbidities, TBLM was positively associated with hip BMD (β_TH BMD_ = 0.36, *P* ≤ 0.001; β_FN BMD_ = 0.26, *P* = 0.01) and cross-section moment of inertia (0.24, *P* ≤ 0.001) and inversely associated with FN FRI (− 0.21, *P* = 0.03) and intertrochanteric FRI (− 0.11, *P* = 0.05) (estimates represent SD difference in bone measures per SD difference in TBLM). Lower limb peak muscle force was positively associated with hip BMD (β_TH BMD_ = 0.28, *P* ≤ 0.001; β_FN BMD_ = 0.23, *P* = 0.008) and inversely associated with FN FRI (− 0.17, *P* = 0.04) and subtrochanteric FRI (− 0.18, *P* = 0.04). Associations of grip strength, gait speed, chair rise time and peak muscle power with hip parameters were close to the null.

**Conclusions:**

Lean mass and lower limb peak muscle force were associated with hip BMD and geometrical FRIs in postmenopausal women. Leg muscle strengthening exercises may therefore help prevent hip fractures in older women.

**Electronic supplementary material:**

The online version of this article (10.1007/s00198-018-4795-z) contains supplementary material, which is available to authorized users.

## Introduction

Sarcopenia, a common disorder of older age characterised by low lean mass plus low muscle strength and/or low physical performance [1], is associated with increased mortality [2] and considerable economic burden [3]. Frailty, one of the main clinical manifestations of sarcopenia, partly reflects functional consequences of impaired muscle strength on physical performance. There has been increasing interest in the effects of sarcopenia and frailty on bone mineral density (BMD) and hip fractures. Physical performance measures which reflect frailty (e.g. grip strength, gait speed, chair rise time) have been shown to predict hip fracture risk in older individuals [4, 5]. Furthermore, low muscle mass has been related to reduced hip BMD [6] and weaker hip strength based on estimates from hip structural analysis [7]. In addition, lower limb muscle strength, assessed using a range of methods, has been related to hip BMD in older populations though it is unclear if these associations are independent of lean mass [6, 8–10].

Few have examined these relationships beyond 70 years of age and thus there is a need for studies exploring the role of lower limb muscle strength in osteoporosis at older ages. We recently demonstrated the feasibility and acceptability of using jumping mechanography to assess lower limb muscle function in a sample of community-dwelling women aged over 70 years old after first excluding women with significant frailty based on their Short Physical Performance Battery (SPPB) score [11]. We subsequently used jumping mechanography estimates to identify lower limb peak muscle force as well as gait speed as two independent predictors of osteogenic impacts in this age group [12].

In the present study, we used cross-sectional data from the Cohort of Skeletal Health in Bristol and Avon (COSHIBA) to investigate the relationship between sarcopenia-related components and osteoporosis in later life. Specifically, we aimed to examine the associations of lean mass and physical performance, including lower limb muscle function assessed by jumping mechanography, with DXA-assessed hip BMD and hip structural analysis-derived measures of hip strength, including whether any associations found were independent of each other. We additionally examined how these physical and muscle performance measures relate to novel geometrical hip fracture risk indices [13–15].

## Methods

### Study population

COSHIBA consists of postmenopausal women recruited during 2007–2009 from primary care registries within Southwest England, and born between 1927 and 1942 [16]. A total of 1064 women were invited to attend research clinic assessments in 2015 and to complete a questionnaire collecting sociodemographic and health data. Full written consent was obtained. Ethical approval was obtained from the South West: Frenchay Research Ethics Committee (14/SW/0138).

### Measurements

#### Dual x-ray absorptiometry

Total body and hip dual x-ray absorptiometry (DXA) scans were collected using a GE Healthcare Lunar Prodigy. Consenting participants who were able to transfer onto the DXA scan bed unaided underwent a total body scan generating fat and lean mass (kg) and left and right hip scans generating total hip and femoral neck BMD (g/cm^*2*^). The manufacturer’s advanced hip structural analysis software was used to derive minimum neck width (mm) and cross-sectional moment of inertia (mm^4^). For the purpose of analyses, the right hip results were used unless there was prior joint replacement, fracture or significant artefact.

#### Hip fracture risk indices

DXA-based finite element analysis was used to derive fracture risk indices for the femoral neck, intertrochanteric and subtrochanteric hip regions. The procedure for calculating hip fracture risk indices using DXA-based finite element analysis has been described previously [13–15]. Briefly, a subject-specific finite element model is automatically constructed from the subject’s hip DXA scan using in-house MATLAB-based programs (The MathWorks, Inc., Natick, MA). The DXA scan is used to generate a proximal femur bone density map and the femur contour. The femur contour is then used to create a two-dimensional finite element mesh, assign material properties (Young’s modulus and yield stress), apply loading/constraint conditions and calculate fracture risk indices (unitless) as the average ratio of von Mises stress to yield stress over the three proximal femur subregions (femoral neck, intertrochanteric and subtrochanteric). The impact force, predicted from the subject’s body weight, height and thickness of hip soft tissue [17], is applied to the greater trochanter; constraint conditions are applied at the femoral head and the distal femur. Greater fracture risk index measurements are associated with greater hip fracture risk independent of other risk factors including femoral neck BMD [15].

#### Grip strength

Grip strength was recorded to the nearest 0.1 kg using a JAMAR digital dynamometer (Patterson Medical, IL), with the handle set at the second point. Three tests were performed on each hand (with 30-s rest between each test) with the participant standing upright (unless unable to stand) with their arm fully extended next to their body and a stiff wrist. The participant was instructed to squeeze as hard as they can for as long as possible, using standard instructions. Measurements alternated between each hand with a total of three attempts for each hand and the maximal value was taken.

#### Gait speed and chair rise time from Short Physical Performance Battery

Gait speed and chair rise time were assessed as part of the Short Physical Performance Battery (SPPB) [18]. Gait speed was assessed using a 4-min timed walk at usual speed, with the best of two attempts scored. Chair rise time was based on the best of 5 timed chair rises without using arms. The SPPB also included tests of side-by-side, semi-tandem, and tandem balances, performed for up to a maximum of 10 s. We also derived a total SPPB score by scoring each of the three tests (i.e. grip strength, chair rise time and standing balance) out of four giving a maximal possible score of 12 [18].

#### European Working Group on Sarcopenia in Older People conceptual stages of sarcopenia

The European Working Group on Sarcopenia in Older People (EWGSOP) conceptual stages of sarcopenia [1] were used to classify women into three groups of no sarcopenia, pre-sarcopenia (low muscle mass without impact on muscle strength or physical performance) and sarcopenia (low muscle mass, plus low muscle strength or low physical performance) or severe sarcopenia (low muscle mass, low muscle strength and low physical performance).

#### Jumping mechanography

Lower limb peak muscle power and force were assessed using a Leonardo Mechanography Ground Reaction Force platform, consisting of two plates with corner sensors that detect voltage proportional to applied force [19]. Sensor recordings were used to derive test-specific performance calculations (Leonardo software version 4.2, Novotec Medical, Germany). Women with an SPPB score ≥ 6 were judged as physically capable and safe to jump and therefore eligible for jumping mechanography tests. Peak power was assessed by two-legged jump and peak force by a one-legged hopping.

#### Heights, weights and comorbidities

Height was measured using a Harpenden stadiometer (Holtain Ltd., Crymych, UK), to the nearest millimetre. Weight was measured using Tanita scales (Tanita UK Ltd., Uxbridge, UK), to the nearest 0.5 kg. Comorbidities were self-reported and grouped as none, one, two or more.

### Statistical analyses

We first examined age-adjusted means of each bone measure across sarcopenia (EWGSOP) and physical performance (SPPB) groups and used an F test to compare the overall difference of the means. Separate linear regression models were then used to examine associations of grip strength, gait speed, chair rise time, total body lean mass and lower limb peak muscle force and power with hip BMD (total hip and femoral neck BMD), hip geometry (cross-sectional moment of inertia, minimum neck width) and hip fracture risk indices (femoral neck, intertrochanteric and subtrochanteric). We fitted two models for each bone parameter; an age-adjusted model (model 1) followed by a model that was additionally adjusted for height, weight (or fat mass instead of weight in the case of lean mass) and comorbidities (model 2).

In additional analyses, the physical performance measures and muscle mass/function variables that remained associated with bone measures after adjustment for covariates (i.e. model 2) were included in mutually adjusted models for each bone measure. To investigate the extent to which total hip BMD explains associations with femoral neck fracture risk index and vice versa, we fitted models with adjustment for total hip BMD and models with adjustment for femoral neck fracture risk index. We examined if lower limb lean mass and appendicular lean mass (calculated as total arms lean mass + total legs lean mass)/height^2^) were more strongly related to hip parameters than total body lean mass by comparing estimates from models with each lean mass measure. Results were presented as difference in SD units in each bone measure per SD unit difference in each physical performance and muscle mass/function measure. All analyses were performed in R (R Foundation for Statistical Computing, Vienna).

## Results

### Participant characteristics

**Fig. 1:**
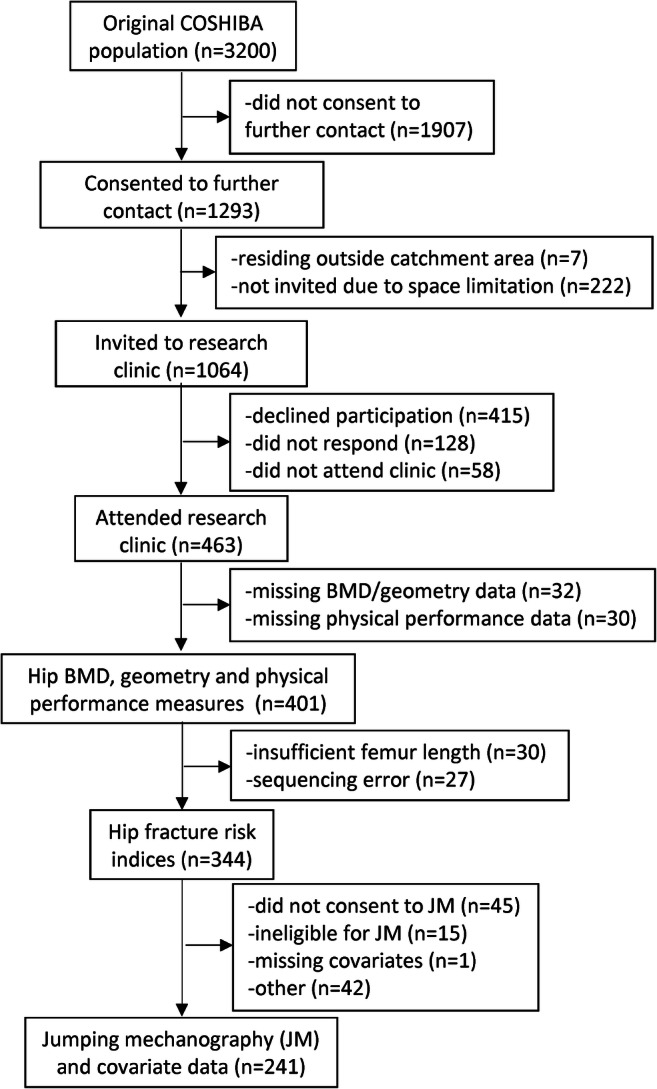
Study flowchart: Cohort of Skeletal Health in Bristol and Avon

A total of 358 women had complete data on hip BMD, geometry and fracture risk indices in addition to measurements to derive EWGSOP and SPPB categories (Online Resource 1). Of these, 241 women had complete data on jumping mechanography and all covariates (Fig. 1, Table 1).

**Table 1:** Characteristics of women from the Cohort of Skeletal Health in Bristol and Avon with data on hip BMD, geometry, fracture risk indices, lean mass, physical performance measures, jumping mechanography measures and all covariates (*n* = 241)

	Mean (SD)
Covariates	
Age (years)	76.4 (2.6)
Height (cm)	158.7 (5.6)
Weight (kg)	66.0 (10.4)
Total body fat mass (kg)	26.6 (7.4)
Comorbidities* [*n*(*%*)]	
None	113 (46.9)
One	101 (41.9)
Two or more	27 (11.2)
Hip density and geometry	
Total hip BMD (g/cm^2^)	0.87 (0.14)
Femoral neck BMD (g/cm^2^)	0.84 (0.13)
Minimum neck width (mm)	30.5 (2.2)
Cross-sectional moment of inertia (mm^4^)	9091.5 (2285.6)
Hip fracture risk indices	
Femoral neck	0.041 (0.027)
Intertrochanteric	0.025 (0.028)
Subtrochanteric	0.004 (0.003)
Lean mass and physical performance measures	
Total body lean mass (kg)	37.4 (4.0)
Grip strength (kg)	21.4 (4.9)
Gait speed (m/s)	1.0 (0.2)
Chair rise time (s)	12.7 (4.2)
Jumping mechanography	
Peak muscle force (kN)	1.3 (0.3)
Peak muscle power (kW)	1.3 (0.3)

*Data for comorbidities shown as *n* (%)

### Mean bone parameters across sarcopenia and physical performance groups

**Fig. 2:**
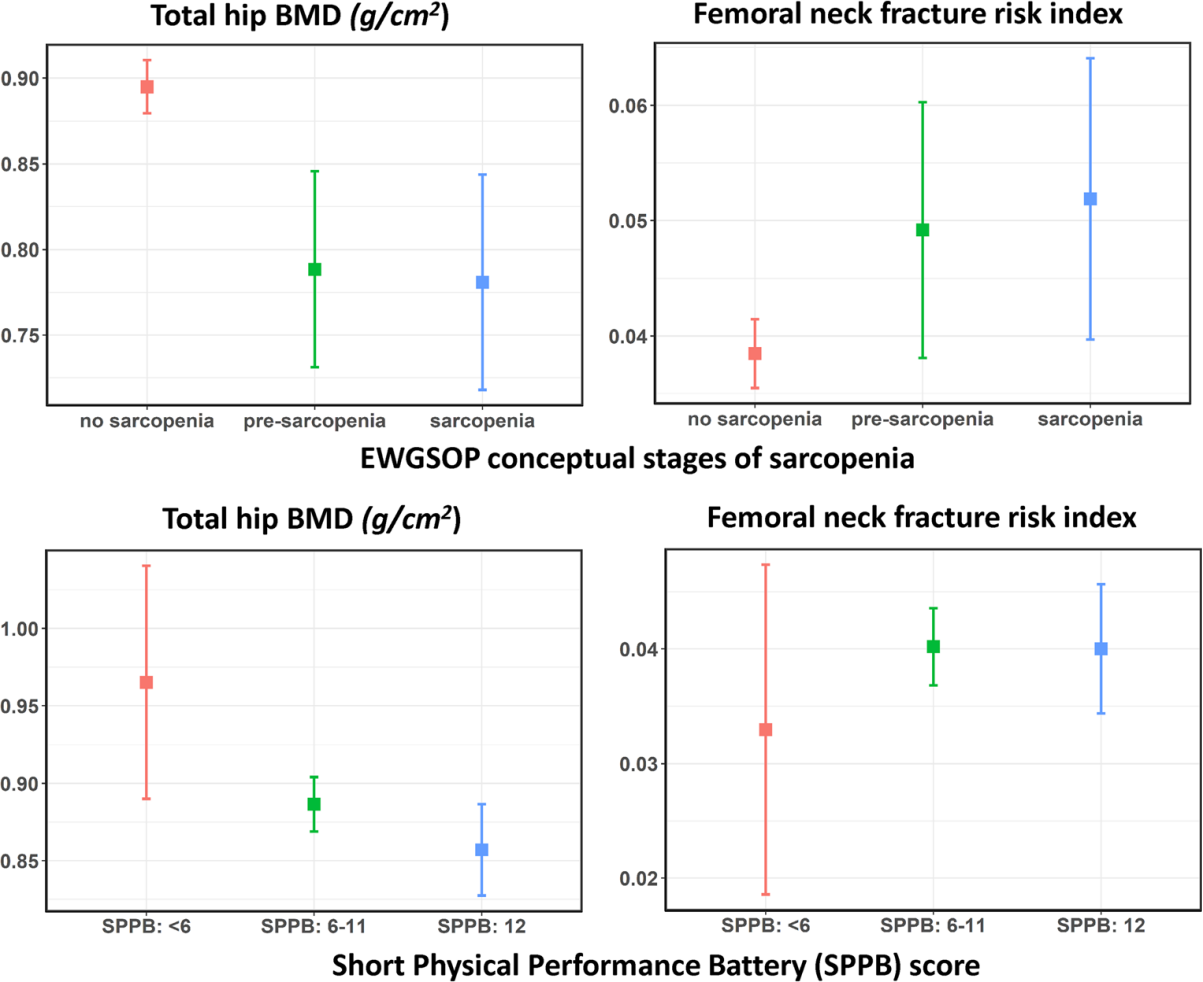
Age-adjusted mean total hip bone mineral density (BMD) and femoral neck fracture risk index across European Working Group on Sarcopenia in Older People (EWGSOP) and Short Physical Performance Battery (SPPB) groups (*n* = 358). EWGSOP stages: no sarcopenia (*n* = 316), pre-sarcopenia (*n* = 23), sarcopenia (*n* = 19). SPPB groups: < 6 (*n* = 14), 6–11 (*n* = 253), 12 (*n* = 91). *P* values from F test comparing overall difference of means were (i) EWGSOP stages: *P* < 0.001 for total hip BMD and *P* = 0.027 for femoral neck fracture risk index, and (ii) SPPB groups: *P =* 0.023 for total hip BMD and *P* = 0.628 for femoral neck fracture risk index

Figure 2 shows age-adjusted mean total hip BMD and femoral neck fracture risk index across EWGSOP and SPPB groups. There was a trend of lower total hip BMD across EWGSOP groups such that the pre-sarcopenia and sarcopenia groups had lower BMD than the group without sarcopenia higher (Fig. 2). A similar trend of higher femoral neck fracture risk index across EWGSOP groups was also observed (Fig. 2). Age-adjusted means for other hip bone parameters are shown in Online Resource 2. There were trends of lower femoral neck BMD and lower cross-sectional moment of inertia and trends of higher intertrochanteric and subtrochanteric fracture risk indices across EWGSOP but not SPPB groups (Online Resource 2). There was little evidence of a difference in minimum neck width across EWGSOP and SPPB groups (Online Resource 2).

### Associations with hip strength

**Fig. 3:**
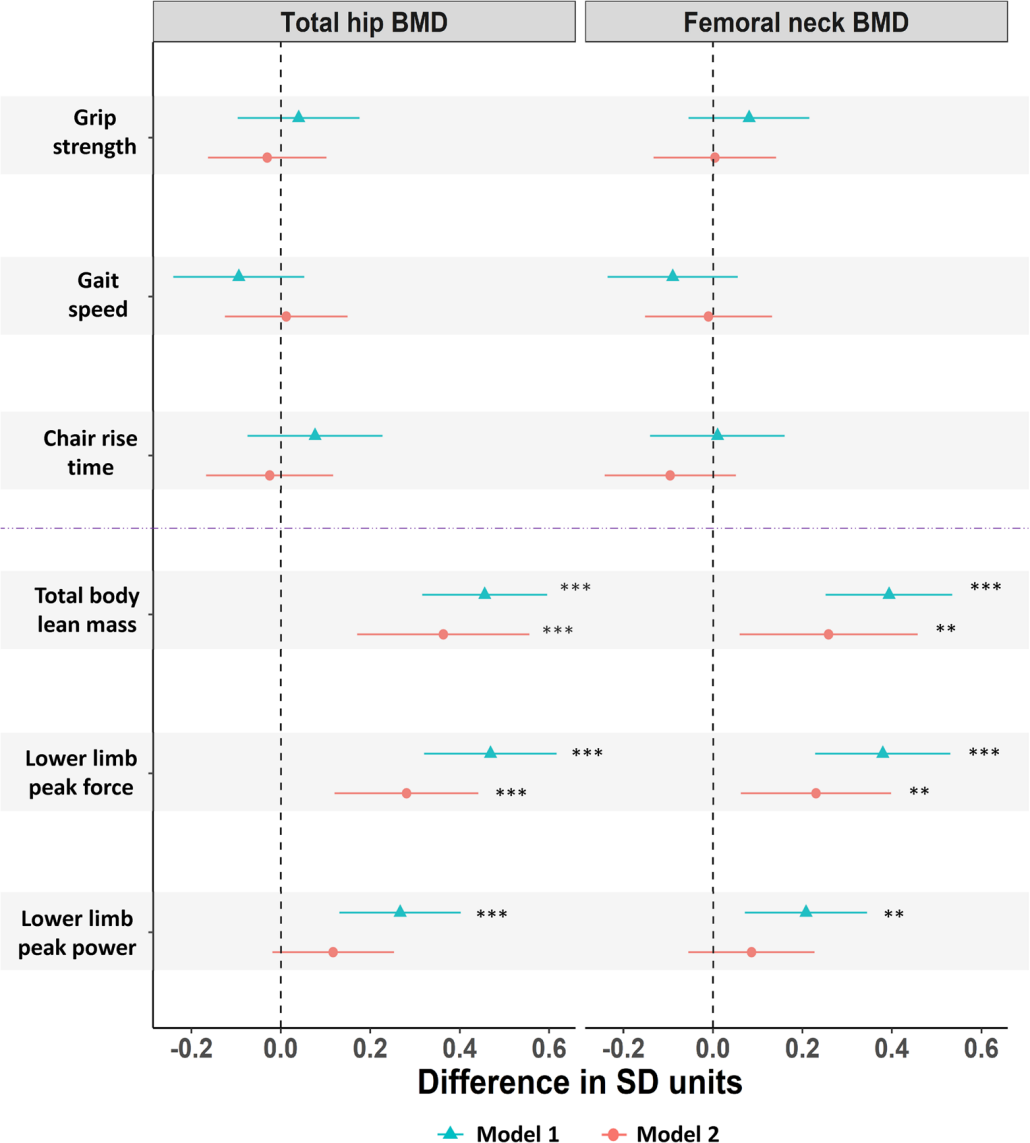
Difference in standard deviation (SD) units in total hip and femoral neck bone mineral density (BMD) per SD unit difference in measures of physical performance and muscle mass/function (*n* = 241). Model 1 adjusted for age. Model 2 adjusted for age, height, weight/fat mass and comorbidities. Asterisk indicates *P* ≤ 0.05, double asterisks indicate *P* ≤ 0.01 and triple asterisks indicate *P* ≤ 0.001. Horizontal bars represent 95% confidence intervals

Figure 3 shows associations of physical performance measures and muscle mass/function with hip BMD. In age-adjusted models, SD unit increases in total body lean mass and lower limb peak muscle force and power were associated with higher total hip BMD and femoral neck BMD (Fig. 3). After further adjustment for height, weight/fat mass and comorbidities, total body lean mass and lower limb peak muscle force remained positively associated with total hip BMD and femoral neck BMD (Fig. 3). Grip strength, gait speed and chair rise time were not associated with hip BMD including both before or after adjustment (Fig. 3).

### Associations with hip geometry

**Fig. 4:**
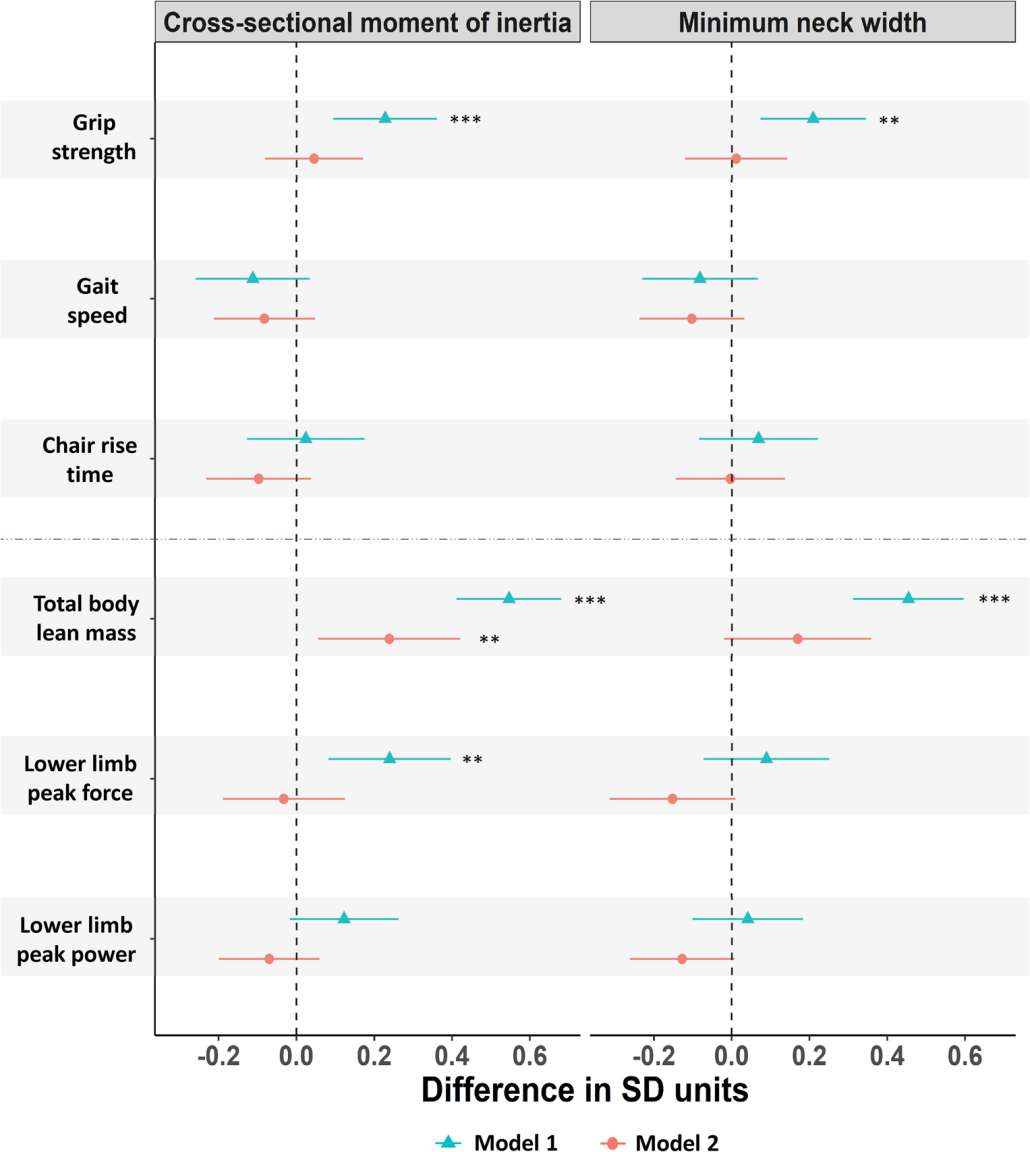
Difference in standard deviation (SD) units in hip cross-sectional moment of inertia and minimum neck width per SD unit difference in measures of physical performance and muscle mass/function (*n* = 241). Model 1 adjusted for age. Model 2 adjusted for age, height, weight/fat mass and comorbidities. Asterisk indicates *P* ≤ 0.05, double asterisks indicate *P* ≤ 0.01 and triple asterisks indicate *P* ≤ 0.001. Horizontal bars represent 95% confidence intervals

Figure 4 shows associations of physical performance measures and muscle mass/function with hip cross-sectional moment of inertia and minimum neck width. In age-adjusted models, SD unit increases in total body lean mass and grip strength were positively associated with both cross-sectional moment of inertia and minimum neck width, whereas lower limb peak muscle force was positively related to cross-sectional moment of inertia only (Fig. 4). After further adjustment for height, weight/fat mass and comorbidities, total body lean mass was positively associated with cross-sectional moment of inertia and marginally associated with minimum neck width (Fig. 4). Lower limb peak muscle force and power were not associated with minimum neck width, and both gait speed and chair rise time were unrelated to cross-sectional moment of inertia and minimum neck width (Fig. 4).

### Associations with hip fracture risk indices

**Fig. 5:**
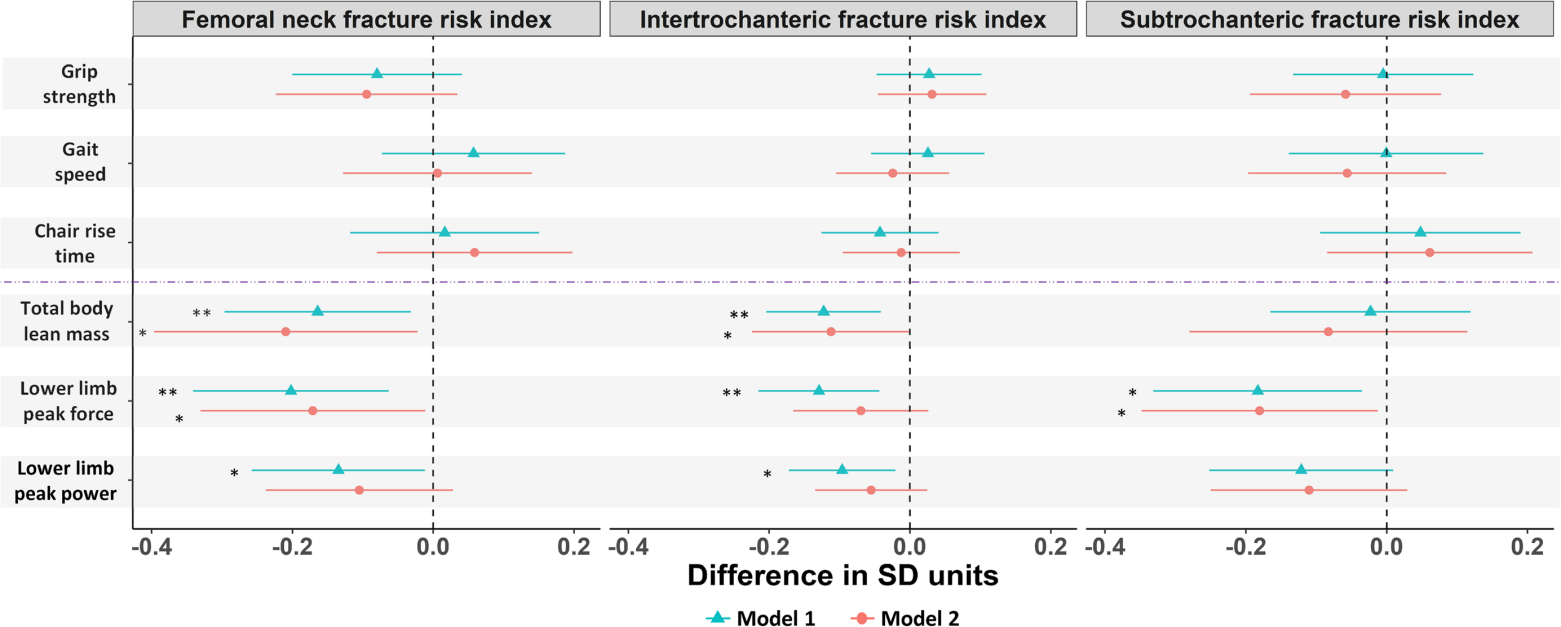
Difference in standard deviation (SD) units in femoral neck, intertrochanteric and subtrochanteric fracture risk indices per SD unit difference in measures of physical performance and muscle mass/function. Model 1 adjusted for age. Model 2 adjusted for age, height, weight/fat mass and comorbidities. P for categorical variables are from test comparing models with and without the measure included. Model 1 unadjusted. Asterisk indicates *P* ≤ 0.05 and double asterisks indicate *P* ≤ 0.01. Horizontal bars represent 95% confidence intervals

Figure 5 shows associations of physical performance measures and muscle mass/function with hip fracture risk indices. In age-adjusted models, SD unit increases in total body lean mass, lower limb peak muscle force and peak muscle power were associated with lower femoral neck and intertrochanteric fracture risk indices, whereas only peak muscle force was associated with subtrochanteric fracture risk index (Fig. 5). After further adjustment for height, weight/fat mass and comorbidities, total body lean mass remained negatively associated with both femoral neck and intertrochanteric fracture risk indices, and lower limb peak muscle force remained negatively associated with both femoral neck and subtrochanteric fracture risk index (Fig. 5). Grip, strength, gait speed and chair rise time were not associated with fracture risk indices (Fig. 5).

### Additional analyses

Models with mutual adjustment for total body lean mass and lower limb peak force (plus all covariates) showed that both measures were independently positively associated with total hip BMD (β_total body lean mass_ = 0.29 (95% CI 0.10 to 0.48), *P* = 0.003; β_peak force_ = 0.29 (0.13 to 0.45), *P* < 0.001) and femoral neck BMD (β_total body lean mass_ = 0.20 (0.00 to 0.40), *P* = 0.053; β_peak force_ = 0.24 (0.07 to 0.41), *P* = 0.006). There was also evidence from these models to suggest that both measures were independently negatively related to femoral neck fracture risk index (β_total body lean mass_ = − 0.17 (− 0.36 to 0.02), *P* = 0.086; β_peak force_ = − 0.17 (− 0.33 to − 0.01), *P* = 0.034).

Total body lean mass and lower limb peak force were no longer associated with femoral neck fracture risk index after adjustment for total hip BMD (β_total body lean mass_ changed from − 0.21 before adjustment to 0.02 (*P* = 0.808) after adjustment for BMD and β_peak force_ changed from − 0.17 before adjustment to 0.01 (*P* = 0.917) after adjustment for BMD), suggesting that hip strength explains their association with fracture risk. Conversely, both measures remained associated with total hip BMD after adjustment for femoral neck fracture risk index (β_total body lean mass_ = 0.22, *P* = 0.004; β_peak force_ = 0.17, *P* = 0.008).

Online Resource 3 presents a comparison of estimates from models using total body lean mass, lower limb lean mass and appendicular lean mass. Overall, these show that broadly similar results were found for each lean mass measure though associations appeared somewhat stronger for total body lean mass (Online Resource 3). Further, the associations of lower limb lean mass with total hip BMD attenuated after mutual adjustment for lower limb peak force (β_lower limb lean mass_ = 0.14, *P* = 0.121), whereas estimates for the latter were unchanged (β_peak force_ = 0.31, *P* < 0.001). For appendicular lean mass index, estimates were slightly attenuated after similar mutual adjustment for lower limb peak force (β_appendicular limb lean mass_ = 0.24, *P* = 0.011).

## Discussion

We examined how grip strength, gait speed, chair rise time, lean mass and lower limb peak muscle force and power relate to hip strength, geometry and novel site-specific geometric hip fracture risk indices in community-dwelling postmenopausal women. Our findings showed that lean mass and lower limb peak muscle force were positively associated with total hip BMD and femoral neck BMD and inversely associated with femoral neck fracture risk index. Lean mass was positively associated with cross-sectional moment of inertia and minimum neck width, and lower limb peak force was negatively related to subtrochanteric fracture risk index. Conversely, none of grip strength, gait speed or chair rise time were associated with hip BMD or fracture risk index, and the associations of grip strength with hip geometry were attenuated in adjusted models.

Our findings that lower limb peak force was more strongly related to hip BMD than peak power contrast those of a previous study of individuals with high bone mass, where peak power (expressed as a ratio with weight) rather than force from jumping mechanography was related to hip BMD [8]. The relatively strong relationship found, which was independent of lean mass, suggests jumping mechanography detects an important component of muscle function with respect to BMD in older women. Given that postmenopausal women are a major high-risk group for hip fracture, and the feasibility of using jumping mechanography in this population, our findings support utility of this method in population studies and might also prove useful for clinical evaluation.

As well as examining associations with both BMD and hip structural analysis-derived variables, we investigated relationships with a novel measure of finite element analysis-derived hip fracture risk index that was previously found to predict hip fracture [13–15]. Peak lower limb muscle force predicted higher hip BMD (total and FN) and lower fracture risk index (femoral neck and subtrochanteric) independently of lean mass, but equivalent relationships were not seen for the primarily hip size-related hip structural analysis-derived variables. Interestingly, the relationship between peak force and fracture risk index was fully explained by differences in BMD, whereas in contrast, the relationship between peak force and BMD was at least partly independent of fracture risk index. These findings suggest that despite the availability of a wide range of derived estimates from hip DXA, BMD remains the most sensitive for detecting relationships with muscle strength. This is in keeping with results from previous trials showing increments in hip BMD following interventions to improve lower limb muscle strength [20–22].

In contrast, we found little association between other measures of physical performance (i.e. grip strength, gait speed and chair rise time), which are strongly linked to premature mortality risk [23], and hip BMD or fracture risk. This may agree with a previous study which found only modest associations between chair rise time and gait speed and hip BMD in older women [24]. Of these measures, only grip strength was positively related to hip cross-sectional moment of inertia and minimum neck width, and although this association attenuated after adjustment, it could agree with a previous study showing that grip strength was the physical performance measure most strongly related to BMD in a sample of physically active postmenopausal women [25].

This study has several important strengths. These include the use of a population-based cohort of community-dwelling over 70-year-old women to investigate the relation between muscle strength and BMD. The use of jumping mechanography provided precise measures of specific elements of muscle function and may represent an important component of muscle function for bone. In addition, we used a novel measure of finite element analysis-derived hip fracture risk index, which aids in the interpretation of our findings with respect to fracture risk. There are also some limitations to this work. Loss of generalisability is likely as we had to exclude some women from jumping mechanography due to frailty. The study was cross-sectional and therefore reverse causation is possible. Residual confounding from unmeasured confounders may also influence the associations found.

In conclusion, our findings showed that lean mass and lower limb peak muscle force were both independently associated with hip BMD and fracture risk indices in postmenopausal women. These findings support the use of interventions to increase leg muscle strength as a means of improving not only hip BMD but also reducing fracture risk. More research may be needed to develop exercise regimes that increase muscle peak force specifically, for example, by incorporating balance exercise and isometric muscle strengthening into a single regime.

## Electronic supplementary material

Online Resource 1 (PDF 430 kb)

Online Resource 2 (PDF 3811 kb)

Online Resource 3 (PDF 248 kb)
