# Correction to: Lean mass and lower limb muscle function in relation to hip strength, geometry and fracture risk indices in community-dwelling older women

**DOI:** 10.1007/s00198-020-05460-7

**Published:** 2020-06-08

**Authors:** A. Elhakeem, A. Hartley, Y. Luo, A. L. Goertzen, K. Hannam, E. M. Clark, W. D. Leslie, J. H. Tobias

**Affiliations:** 1 Musculoskeletal Research Unit, Translational Health Sciences, Bristol Medical School, University of Bristol, Bristol, UK; 2 MRC Integrative Epidemiology Unit, Population Health Sciences, Bristol Medical School, University of Bristol, Bristol, UK; 3 Department of Mechanical Engineering, University of Manitoba, Winnipeg, Canada; 4 Department of Radiology, University of Manitoba, Winnipeg, Canada; 5 Department of Internal Medicine, University of Manitoba, Winnipeg, Canada

**Correction to: Osteoporosis International (2019) 30:211–220**

10.1007/s00198-018-4795-z

The original version of this article, published on 14 December 2018, unfortunately contained a mistake.

The article was originally published under a CC BY-NC 4.0 license, but has now been made available under a CC BY 4.0 license.

The PDF and HTML versions of the paper have been modified accordingly.

## Open Access

This article is licensed under a Creative Commons Attribution 4.0 International License, which permits use, sharing, adaptation, distribution and reproduction in any medium or format, as long as you give appropriate credit to the original author(s) and the source, provide a link to the Creative Commons licence, and indicate if changes were made. The images or other third party material in this article are included in the article's Creative Commons licence, unless indicated otherwise in a credit line to the material. If material is not included in the article's Creative Commons licence and your intended use is not permitted by statutory regulation or exceeds the permitted use, you will need to obtain permission directly from the copyright holder. To view a copy of this licence, visit http://creativecommons.org/licenses/by/4.0/.

