# Supplementary material for: Lean mass and lower limb muscle function in relation to hip strength, geometry and fracture risk indices in community-dwelling older women
Source: Osteoporos Int. 2018 Dec 14;30(1):211–20. doi: 10.1007/s00198-018-4795-z (PMC6331743; doi:10.1007/s00198-018-4795-z)
Supplement: Supplementary file 1 — (PDF 430 kb) [file 198_2018_4795_MOESM1_ESM.pdf]

Online Resource 1 Characteristics of women from the Cohort of Skeletal Health in Bristol and Avon with data on hip BMD, geometry and fracture risk indices, the European Working Group on Sarcopenia in Older People (EWGSOP) conceptual stages of sarcopenia, and the Short Physical Performance Battery (n=358).

age (years) [mean (SD)]	76.7 (2.9)
Hip density and geometry [mean (SD)]	
total-hip BMD (g/cm^2)	0.88 (0.15)
femoral neck BMD (g/cm^2)	0.85 (0.14)
minimum neck width (mm)	30.5 (2.2)
CSMI (mm^4)	9210.6 (2454.4)
Hip fracture risk indices [mean (SD)]	
femoral neck	0.040 (0.028)
intertrochanteric	0.024 (0.028)
subtrochanteric	0.004 (0.003)
EWGSOP [N (%)]	
no sarcopenia	316 (88.3)
pre-sarcopenia	23 (6.4)
sarcopenia/severe sarcopenia	19 (5.3)
SPPB score [N (%)]	
<6	14 (3.9)
6-11	253 (70.7)
12	91 (25.4)
