# Supplementary material for: Lean mass and lower limb muscle function in relation to hip strength, geometry and fracture risk indices in community-dwelling older women
Source: Osteoporos Int. 2018 Dec 14;30(1):211–20. doi: 10.1007/s00198-018-4795-z (PMC6331743; doi:10.1007/s00198-018-4795-z)

Online Resource 2 Age-adjusted mean femoral neck bone mineral density (BMD), cross-sectional moment of inertia, minimum neck width, and intertrochanteric and subtrochanteric fracture risk indices across EWGSOP and SPPB groups (n=358). P-values were from F test comparing overall difference of means.

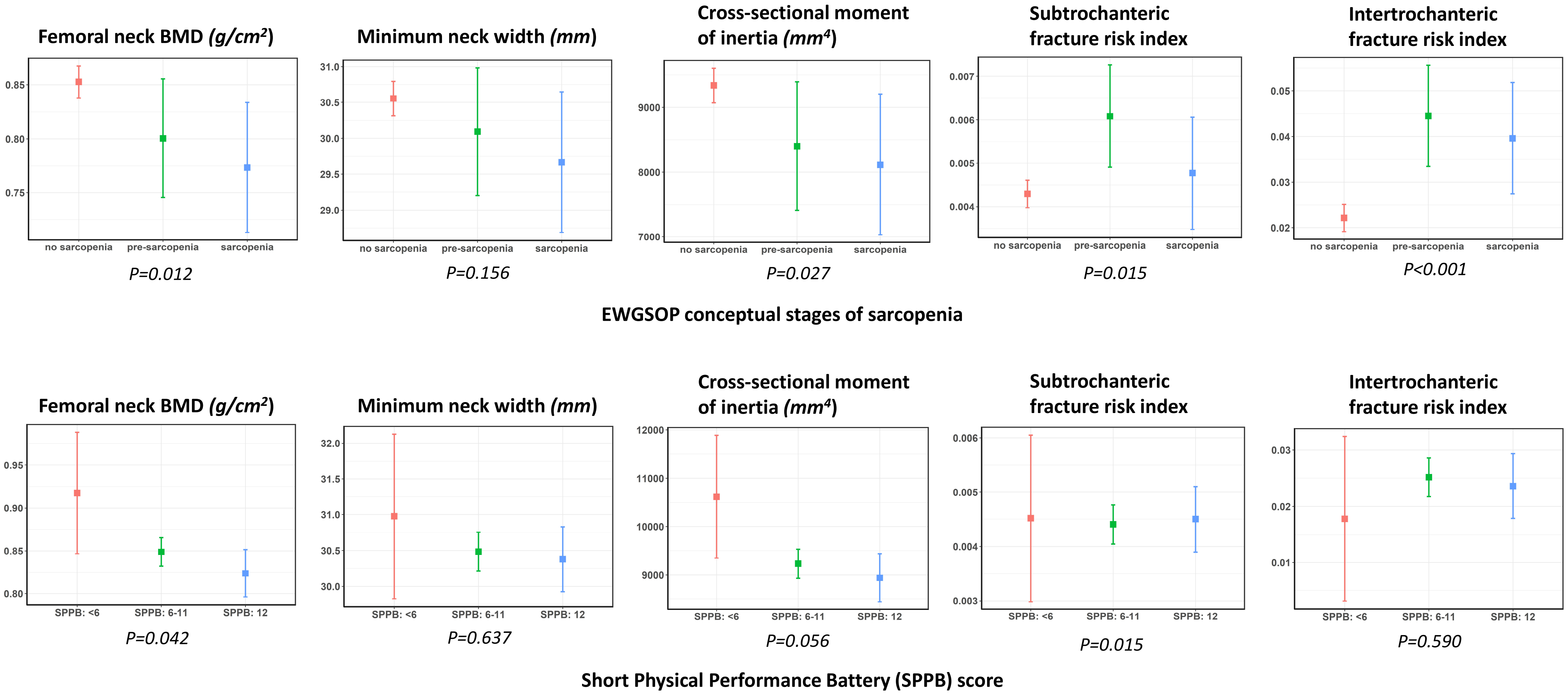

Supplement: Supplementary file 2 — (PDF 3811 kb) [file 198_2018_4795_MOESM2_ESM.pdf]
