# Supplementary material for: Lean mass and lower limb muscle function in relation to hip strength, geometry and fracture risk indices in community-dwelling older women
Source: Osteoporos Int. 2018 Dec 14;30(1):211–20. doi: 10.1007/s00198-018-4795-z (PMC6331743; doi:10.1007/s00198-018-4795-z)
Supplement: Supplementary file 3 — (PDF 248 kb) [file 198_2018_4795_MOESM3_ESM.pdf]

Online Resource 3 Difference in standard deviation (SD) units in each bone parameter per SD unit difference in total body lean mass, lower limb lean mass and appendicular lean mass index (n=241).

	Model 1<br>β (95% CI)	*P*	Model 2<br>β (95% CI)	*P*
Total hip BMD				
total body lean mass	0.455 (0.316 to 0.595)	<0.001	0.363 (0.170 to 0.555)	<0.001
appendicular lean mass index	0.483 (0.341 to 0.625)	<0.001	0.304 (0.121 to 0.487)	0.001
lower limb lean mass	0.358 (0.241 to 0.475)	<0.001	0.207 (0.034 to 0.381)	0.020
Femoral neck BMD				
total body lean mass	0.393 (0.251 to 0.534)	<0.001	0.258 (0.059 to 0.457)	0.012
appendicular lean mass index	0.338 (0.190 to 0.485)	<0.001	0.175 (-0.016 to 0.366)	0.073
lower limb lean mass	0.287 (0.167 to 0.406)	<0.001	0.104 (-0.075 to 0.282)	0.256
Cross-sectional moment of inertia				
total body lean mass	0.546 (0.411 to 0.680)	<0.001	0.238 (0.056 to 0.420)	0.011
appendicular lean mass index	0.278 (0.127 to 0.429)	<0.001	0.052 (-0.142 to 0.246)	0.600
lower limb lean mass	0.403 (0.288 to 0.518)	<0.001	0.108 (-0.055 to 0.271)	0.195
Minimum neck width				
total body lean mass	0.453 (0.311 to 0.595)	<0.001	0.169 (-0.020 to 0.358)	0.081
appendicular lean mass index	0.133 (-0.022 to 0.289)	0.095	-0.030 (-0.232 to 0.173)	0.773
lower limb lean mass	0.333 (0.213 to 0.453)	<0.001	0.094 (-0.074 to 0.263)	0.273
Femoral neck fracture risk index				
total body lean mass	-0.164 (-0.296 to -0.032)	0.016	-0.209 (-0.397 to -0.022)	0.029
appendicular lean mass index	-0.200 (-0.334 to -0.065)	0.004	-0.192 (-0.369 to -0.015)	0.035
lower limb lean mass	-0.116 (-0.227 to -0.006)	0.040	-0.120 (-0.287 to 0.047)	0.161
Subtrochanteric fracture risk index				
total body lean mass	-0.023 (-0.165 to 0.119)	0.751	-0.083 (-0.280 to 0.115)	0.412
appendicular lean mass index	-0.193 (-0.337 to -0.050)	0.009	-0.178 (-0.366 to 0.011)	0.066
lower limb lean mass	-0.032 (-0.150 to 0.087)	0.601	-0.045 (-0.220 to 0.130)	0.612
Intertrochanteric fracture risk index				
total body lean mass	-0.123 (-0.204 to -0.041)	0.003	-0.112 (-0.224 to 0.000)	0.052
appendicular lean mass index	-0.173 (-0.255 to -0.091)	<0.001	-0.110 (-0.216 to -0.003)	0.044
lower limb lean mass	-0.104 (-0.172 to -0.037)	0.003	-0.068 (-0.168 to 0.032)	0.183

Model 1 adjusted for age. Model 2: adjusted for age, height (except for appendicular lean mass index), fat mass and comorbidities.
